# The Quality of Puhui Early Education Services in Chinese Teachers’ Perspectives: Evidence from a National Validation Study

**DOI:** 10.3390/ijerph191710870

**Published:** 2022-08-31

**Authors:** Yifang Wang, Yong Jiang, Hui Li, Yu Zhou, Xinxin Liu

**Affiliations:** 1 Shanghai Institute of Early Childhood Education, Shanghai Normal University, Shanghai 200234, China; 2 Institute of Early Childhood Education, Faculty of Education, East China Normal University, Shanghai 200062, China; 3 Macquarie School of Education, Macquarie University, Sydney, NSW 2109, Australia

**Keywords:** Puhui early education service, service quality, instrument development, teacher evaluation, China

## Abstract

In this study, we examined the quality of Puhui early education services perceived by Chinese teachers with evidence from a national validation study. Firstly, using a stratified cluster sampling method, the Puhui Early Education Service Quality Scale (PEESQ) was developed and validated with 994 Chinese teachers. Secondly, the psychometric properties indicated that PEESQ is a reliable and valid scale with three dimensions: service adequacy, service balance, and public service welfare. Thirdly, descriptive statistics found that Chinese teachers generally appreciated the quality of Puhui services, ranking the construct of “public welfare” the highest and “service balance” the lowest. Finally, hierarchical regression analysis found that urbanicity positively predicted teacher evaluations after controlling for demographic and kindergarten factors. The findings imply that more funding support and policy attention should be provided to help enhance the quality of Puhui early education services (EES). Moreover, this study extends the framework of research on teacher evaluation of EES and provides useful tools to evaluate universal EES in China and beyond.

## 1. Introduction

China has been suffering from the “3A” problems of early education for decades. There are not enough kindergartens to serve the number of children who require them (accessibility), parents cannot afford tuition fees (affordability), and the services are of poor quality (accountability) [1,2]. To thoroughly solve the “3A” problems, the central government designed and launched the “Puhui” early education service (EES) system in 2010, subsidizing all public and some private kindergartens (ages 3–6) to provide an affordable and accountable service [3]. This educational innovation substantially enhanced the gross enrollment rate for EES from 50.9% in 2009 to 88.1% in 2021 [4,5]. However, this “great leap forward” in the quantity of EES does not necessarily translate into the enhancement of the quality of Puhui EES. Some studies explored the satisfaction of service receivers (i.e., parents and the public) and found that Chinese parents were mostly satisfied with Puhui EES [3,6], but no research has yet evaluated the service providers’ (i.e., teachers and principals) perceived quality of Puhui EES, which is more important for service improvement. To address this gap, we developed and validated a national scale to conduct a service quality evaluation with a large-scale sample of Chinese teachers.

## 2. Literature Review

### 2.1. The Quality of Puhui Early Education Service

Globally, international organizations and countries are working to improve EES’s affordability, availability, quality, and equity to enable as many families and children as possible to enjoy high-quality early education [7,8,9]. Many countries have implemented universal provision programs, accessible to children who meet age eligibility criteria, emphasizing the potential benefits for all children and the greater impact on disadvantaged children, such as the Universal Children Care Benefit and the Universal Child Care Program [10,11]. These programs are concerned with the equity of children’s access to EES, for such access is presented as a right of all children.

In China, the emergence of the Puhui EES is closely related to the social welfare and economic systems. On the one hand, the moderately inclusive shift of the Chinese social welfare system provides a suitable foundation and conditions for Puhui EES. On the other hand, to cope with the aging population crisis and increase the birth rates, the central government of China has highly prioritized the development of universal EES since 2010. In particular, the *State Council’s Opinions on the Current Development of Early Childhood Education* (hereafter “the opinions”) were enacted to establish a national Puhui EES system [12]. This system demands that governments at all levels subsidize and facilitate the development of affordable EES, aiming to solve the “3A” problems thoroughly [1]. Accordingly, all public and most private kindergartens were included in the Puhui EES [13]. In Chinese, Puhui (普惠) means “universally and generally involving all people with some beneficial, favorable, and kind offer”, and should be fairly good and inexpensive. Therefore, Puhui EES should be “universal, affordable, accessible, and accountable” for all needy families of young children aged 3 to 6 years [3]. Since the Puhui policy was implemented over a decade ago, the gross enrollment rate for EES jumped from 50.9% in 2009 to 88.1% in 2021 [4,5]. However, such data cannot fully reflect the effectiveness of the Puhui EES. Furthermore, a decade ago in China, most parents only wanted their children to “be able to enter a school”; more recently, parents have raised their standards and now want their children to “access…a high-quality school” [14]. Therefore, the focus of the Puhui EES has shifted from expanding coverage to improving the service quality.

To date, Chinese scholars have debated the definition of Puhui EES quality from various perspectives. Some define the Puhui EES quality as the degree of meeting young children’s developmental needs [15,16]. Some extend this definition by adding the need to entertain other stakeholders such as the government, community, organizers, parents, and teachers [17]. From our perspective, the quality of Puhui EES refers to the degree of being “affordable” and “accountable” in meeting young children’s educational needs. Accordingly, we need to develop a systematic and comprehensive scale to evaluate the “affordability” and “accountability” of Puhui EES. To develop this scale, we must generate the performance indicators of “affordability” and “accountability” of Puhui EES. In addition to the affordability–accountability constructs, the “structure–process” dichotomy has been extensively employed to evaluate the quality of EES [18]. Structural quality refers to facilities/resources, staff-to-child ratios, and staff qualifications. Process quality focuses on the interactions between the child and teacher, child and child, teacher and parent, and teacher and teacher. Adopting this framework, the Organization for Economic Co-Operation and Development identified seven aspects of quality: (1) interaction or process quality, (2) child outcome quality, (3) standards about parent/community outreach and involvement, (4) orientation quality, (5) structural quality, (6) educational concept and practice, and (7) operational quality. These aspects can be categorized under the two aforementioned types of quality, structure and process quality [7]. Aspects (1) to (3) are concerned with process quality, whereas (4) to (7) focus on structural quality. In China, the “input–output” model has also been adopted to develop quality indicators such as hardware input, software input, ontology output, functional output, and benefits [14,16]. Accordingly, the quality of Puhui EES has improved since 2010. However, the quality of overall EES (including non-Puhui services) is still lower than that of elementary education.

The above review indicates that Puhui EES quality has been evaluated based on affordability, accountability, structural quality, process quality, input quality, and output quality. However, there is no systematic approach that considers the policies and contexts of Puhui, preventing us from comprehensively measuring Puhui EES quality. Therefore, we proposed incorporating them into a holistic evaluation system with new constructs: service adequacy at the input level, service balance at the distribution level, and public service welfare at the output level. Accordingly, in this study, service adequacy mainly refers to the resource investment adequacy of the Puhui EES, including software and hardware, such as the child–teacher ratio. Meanwhile, service balance mainly refers to the resource distribution balance of the Puhui EES, including rural–urban and inter-kindergarten support, such as increasing financial support for rural kindergartens. Finally, public service welfare mainly refers to the output benefits to children and society of the Puhui EES, including personal and social benefits, such as providing family parenting. This three-construct framework is suitable for evaluating Puhui EES quality because it is consistent with the Puhui policy goals and focuses on the rooted problems of EES. This framework is based on the Input–Output–Outcome (IOO) model and the Service Quality (SERVQUAL) model [19,20]. Specifically, the IOO model connects the input, process, output, and result, reflecting the transformation and utilization of resources. It has been widely used in service quality evaluation and is associated with the logic model. In addition, the SERVQUAL model is mainly based on the difference theory, whose theoretical core is the Service Quality Gap Model (also called the Expectation Perception Model). The SERVQUAL model has five constructs: tangibility, reliability, responsiveness, assurance, and empathy. For example, based on the SERVQUAL model, a scholar constructed the service quality evaluation indicator of community education information, applying the perceived service quality quadrant to analyze and interpret the index importance to make the system more feasible [21]. We believe that this three-construct framework, based on the IOO and SERVQUAL models, could be developed into an evaluation tool of the quality of Puhui EES.

### 2.2. Service Quality Evaluation by Teachers

Teachers are viewed as the most valuable stakeholders when evaluating the quality of EES, including the Puhui EES. First, they are the service providers and play an active supporting/shaping role in quality assurance [22]. Second, their professionalism and performance, competency, and enthusiasm are critical to delivering quality services. Third, teacher evaluation could provide authentic evidence to judge the quality of Puhui EES [23]. Therefore, this study is dedicated to gathering teachers’ insights and self-evaluation of the quality of Puhui EES as insiders. In China, a previous study by Jiang and Pang interviewed kindergarten teachers and found that they highly valued the affordability of Puhui EES [24]. However, no studies have asked Chinese teachers to systematically evaluate the quality of Puhui EES because of the lack of reliable instruments. To address this gap, we developed and validated a scientific scale to evaluate service quality with Chinese teachers.

Teachers’ evaluations might be influenced by the program design, principal leadership, and their backgrounds [25]. For example, the central control of curriculum was a significant factor influencing Cypriot and English teachers’ evaluations [26]. Teacher’s age was also an important factor: preschool teachers aged 19–29 were more accepting of male teachers than other age groups, indicating that young teachers tended to agree with more progressive opinions than older teachers [27]. In China, the stability and rationality of program content and implementation, faculty benefits, and teachers’ teaching experience and backgrounds were identified as key factors [28,29]. In addition, the urbanicity and ratings of kindergarten might also affect teachers’ quality evaluation [3,6], but their real impact has not been confirmed in the Chinese context. In particular, China features a national rating system of kindergartens’ quality: exemplary, first-class, second-class, and third-class kindergartens. The exemplary kindergarten has the best quality, whereas the third class has the lowest. Furthermore, the *Measures for Supervision and Evaluation of Kindergarten Running Behavior* mandate regular quality evaluation by the local educational authorities, focusing on the hardware and software of each kindergarten [30]. In sum, teachers’ demographics and kindergarten attributes might influence teachers’ evaluations in multiple ways. In this study, we explore the above factors influencing teachers’ evaluation of the quality of Puhui EES.

### 2.3. The Context of This Study

While the Puhui EES has significantly progressed since 2010, many problems remain. For example, the quantity and quality of kindergarten teachers in lower-rated kindergartens lag behind those of high-rated ones [3,6]. Moreover, the urban–rural gaps might have exacerbated the diversity of the Puhui EES quality and thus have caused the growing opportunity gaps between urban and rural children [17]. To understand these developed–developing and rural–urban gaps, we must conduct a national survey study of the Puhui EES quality. Accordingly, we developed and validated a reliable scale for teachers’ service quality evaluation and explored the influential factors. In particular, the following questions guided this national study:

1. What are the psychometric properties of the service quality evaluation tool?
2. How do Chinese teachers view the quality of Puhui early education services?
3. What are the factors influencing Chinese teachers’ quality evaluation?

## 3. Materials and Methods

### 3.1. Participants

Stratified cluster sampling was conducted to recruit kindergarten teachers for this national survey study. First, we randomly selected three provinces to represent eastern, central, and western China: Shandong, Jiangxi, and Guizhou, respectively. Overall, the economic and ECE development levels of Shandong Province are relatively high, followed by Jiangxi Province and Guizhou Province. Second, we randomly sampled one city from each participating province, resulting in three participating cities: Qingdao, Shangrao, and Guiyang. Qingdao and Guiyang are both capital cities, and Shangrao is a relatively developed city in Jiangxi Province. These three cities have made great efforts and progress in developing Puhui EES. Third, we randomly sampled ten kindergartens from each city and distributed 1023 online questionnaires to their teachers. Fourth, 994 questionnaires were completed and returned, resulting in a high return rate of 97.2%. The high return rate was because the local government distributed and collected the questionnaires. Coincidently, there were no significant differences in the cities’ return rates (less than 1%).

The sample of this study covered the countryside (8.0%), villages (20.4%), counties (48.0%), and urban cities (23.6%) as administratively classified in China. According to the Department of Development Planning and Ministry of Education statistics, the ratio of urban to rural kindergarten teachers was about 3:7. Thus, the proportions of kindergarten teachers from the rural and urban areas in this study were close to this national norm. Their education levels ranged from junior high or below (4.2%), senior high (38.3%), junior college (42.5%), and undergraduate or above (15.0%). Since 2010, all public kindergartens have been included in the Puhui EES because they are commonly owned and sponsored by governmental organizations and tend to be affordable and accountable for all families in need. However, private kindergartens can be classified into two groups, Puhui and non-Puhui private kindergartens, based on whether they are subsidized by governments and provide affordable and quality services. Thus, most participants were from public kindergartens (80.0%), with a few from Puhui private kindergartens (20.0%). In particular, most of the participants were from exemplary kindergartens (69.2%), followed by first-class kindergartens (18.4%), second-class kindergartens (6.9%), and third-class kindergartens (5.5%). The participants’ demographic information is presented in Table 1.

### 3.2. Puhui Early Education Service Quality Scale (PEESQ)

The three-construct framework was adopted to develop the quality indicators for the service quality evaluation: service adequacy, service balance, and public service welfare. First, we extensively analyzed relevant policy documents and developed quality indicators of Puhui EES, grouping the items into three dimensions. Second, we consulted nine experts through three rounds of Delphi expert interviews to examine and validate the quality indicators. Some items were deleted based on the experts’ feedback. The chosen 34 items were classified into three dimensions. The first and the second authors reviewed and revised the 34 items to finalize the first draft of the instrument. Then, convenience and purposeful sampling were conducted to recruit 111 kindergarten teachers from seven provinces for the pilot study. They were asked to complete the survey online, providing feedback on the items’ wording and identifying any problems. Their feedback was collected and examined, and the corresponding items were refined to improve clarity and readability. The final version includes 32 items that can be classified into three dimensions (see Appendix A for the scale).

The Puhui Early Education Service Quality Scale (PEESQ) has two parts. The first part is a demographic survey collecting information about the respondents’ age, teaching experience, educational background, job position, school tenure, urbanicity, kindergarten type, and rating. The second part is the PEESQ, which comprises 32 items rated on a 5-point Likert scale (1 = extremely disagree to 5 = extremely agree; ordinary type) based on the participants’ judgments of and feelings about Puhui EES quality. Specifically, service adequacy contains seven items (e.g., child–teacher ratio, percentages of degree holders), service balance contains 12 items (e.g., expanding Puhui resources in rural areas, financial support for rural kindergartens), and public service welfare contains 13 items (e.g., effective teacher–child interaction, provide family parenting). The survey was conducted using the written form of Mandarin Chinese. For instance, item 1, “The child–teacher ratio meets standards”, belongs to the service adequacy construct and was written in Chinese as “幼儿和教师的配比符合标准”. The statistical means of each item, dimension, and construct were taken as the final scores. A high score indicates a high evaluation and a positive perception of the Puhui EES quality in this study.

### 3.3. Procedures

Ethical procedures were followed throughout the study. First, the study was approved by the ethics committee of the corresponding author’s university. Second, consent was obtained from the local governments, educational directors, and the principals of the participating kindergartens. Third, kindergarten teachers were briefed about the survey via an introduction at the beginning of the questionnaire. Finally, they were considered to consent to participate if they completed and returned the questionnaire.

The survey scale was created and collected online using www.wjx.cn, the leading online survey platform in China. All survey data were analyzed using IBM SPSS 26.0 (IBM, Armonk, NY, USA) and MPLUS 8.0 (Mplus Software, Jakarta, Indonesia). First, data cleaning was conducted to replace very few missing values (<0.5%) with the mean of the concerned variable. Next, the psychometric properties were analyzed using item analysis, exploratory factor analysis (EFA), and confirmatory factor analysis (CFA) to explore and verify the construct validity and reliability of the scale. Descriptive statistics were then employed to explore teachers’ overall evaluations of Puhui EES quality. Last, hierarchical regression analyses were conducted to examine the main effects and predictors.

## 4. Results

### 4.1. Psychometric Properties of PEESQ

Item analysis was adopted to test the adequacy of 32 items. After analyzing the items by extreme group testing, the item total correlation method, and internal consistency reliability value testing, we decided to keep them all. The reasons are as follows: (1) the *CR* value of each item was greater than 3.00, indicating good discrimination ability; (2) the correlation coefficients between items and the total score are between 0.63 and 0.88, greater than 0.40; (3) the Cronbach’s α coefficient of the scale was 0.981, above 0.7 [31,32,33].

EFA was conducted using SPSS version 26.0 on the first half (*n* = 497) of the sample to disclose the structure of PEESQ. First, the adaptability of the data was tested, and the fit for EFA was confirmed [KMO = 0.98, Bartlett spherical test *χ*^2^ = 19,059.03 (*df* = 496, *p* < 0.001)]. Then, a three-factor model was produced for the scale that can explain 72.39% of the total variation using principal axis factoring (PAF) with a direct oblimin method, indicating satisfactory construct validity. The eigenvalues of the three dimensions were 20.50, 1.37, and 1.30, respectively. The factor load of the item was between 0.69 and 0.89, all greater than 0.45; the degree of commonality was between 0.56 and 0.81, all greater than 0.2; and the common factor of the factor composition structure was also relatively stable. Accordingly, there was no need to delete any items. Finally, we adjusted the items according to the results of the exploratory factor analysis in Table 2. The variance explanation rate of the first factor was 4.05%, and the seven items included were all from the service adequacy construct, mainly referring to the investment of the Puhui EES; therefore, it was still named “service adequacy”. The variance explanation rate of the second factor was 4.27%. Most of the 12 items included were from the service balance construct, and some were from the original service adequacy and public service welfare constructs. Most of them refer to kindergarten support and urban and rural support; this factor was named “service balance”. The variance explanation rate of the third factor was 64.07%, and all 13 items were from the original public service welfare construct that referred to personal and social benefits. Therefore, it was named “public service welfare”. As a result, the PEESQ was confirmed to have three dimensions: service adequacy, service balance, and public service welfare.

**Figure 1:**
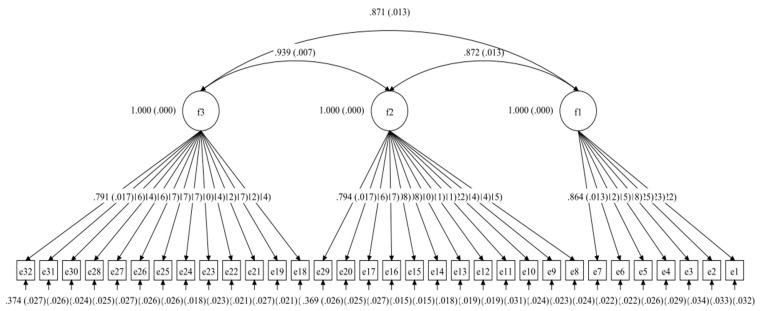
CFA, confirmatory factor analysis; PEESQ, Puhui Early Education Service Quality Scale. Model fit: *χ*^2^ = 984.22, *df* = 360, *χ*^2^/*df* = 2.73, *p* < 0.000, CFI = 0.97, TLI = 0.96, SRMR = 0.05, RMSEA = 0.02 (90% CI [0.042, 0.043]), AIC = 92,262.40, BIC = 93,040.67.

CFA analysis was conducted using MPLUS version 8.0 to confirm the three-factor structure that the EFA result suggested with the second half (*n* = 497) of the data. Considering all items as continuous data, we used maximum likelihood estimation. The factor loads of the 32 items ranged from 0.61 to 0.93, and all reached a significant level (*p* < 0.001), indicating that the PEESQ has a good factor structure. As shown in Figure 1, the *χ*^2^/*df* of this scale was 2.73, which is less than 5, and the value of RMSEA was 0.02, which is less than 0.05 [34]; the values of CFI and TLI were 0.97 and 0.96, which are greater than 0.90 [35]; the value of SRMR was 0.05, which is less than 0.08 [36]. Thus, the CFA results confirmed the construct validity.

As shown in Table 3,the Cronbach’s α value for the PEESQ was 0.98, which is above 0.90, and the reliability of each factor ranged between 0.91 and 0.97; therefore, the entire scale was determined to have high reliability. Meanwhile, the split-half reliability ranged between 0.80 and 0.90, indicating that the internal consistency reliability ranged from excellent to acceptable [37]. In addition, the correlation matrix between three dimensions ranged from 0.69 to 0.77 (*p* < 0.000), indicating acceptable reliability.

### 4.2. Descriptive Analysis of Teachers’ Evaluation of the Quality of Puhui EES

The Puhui EES quality was recognized by the majority (75.8%) of Chinese teachers, with the means of the overall score being higher than 4.0 (*M* = 4.41, *SD* = 0.63). Specifically, teachers exhibited highly positive evaluations of public service welfare (*M* = 4.50, *SD* = 0.61), followed by service adequacy (*M* = 4.46, *SD* = 0.64) and service balance (*M* = 4.29, *SD* = 0.75). Among the 32 items, the two with the lowest mean scores were “teacher salary” (*M* = 3.90, *SD* = 0.59) and “educational funding” (*M* = 4.06, *SD* = 0.62), indicating relatively low satisfaction with financial investment.

### 4.3. Factors Affecting Teachers’ Quality Evaluation

Spearman correlation analysis was conducted to explore the variables correlated with teachers’ evaluations of Puhui EES quality. As shown in Table 4, the following variables were found to significantly correlate with teachers’ total evaluation scores (*ps* < 0.001): age (<20, 20–29, 30–39, ≥40), educational background (junior high or below, senior high, junior college, undergraduate or above), school tenure (yes, no), job position (ordinary, monitor, grade leader, director of nursing and teaching, principal), kindergarten rating (exemplary, first-class, second-class, third-class kindergarten), and urbanicity (countryside, village, county, and urban city).

We performed a three-step hierarchical regression analysis to identify the predictors of teacher evaluation. The significantly correlated variables were entered in three steps. In step 1, age and educational background were entered to control the influence of demographic variables. In step 2, kindergarten variables, including job position, school tenure, and kindergarten rating, were entered to explore the influence of kindergarten attributes on teacher evaluation. Finally, in step 3, we entered the urbanicity factor, countryside/villages and county/urban city, which is directly correlated with teacher evaluation.

The changes in *R*^2^ between the three steps indicated three key findings. First, demographic variables can only jointly explain 1.6% of the total evaluation scores, the lowest percentage in this study. Age and educational background were negative predictors of teachers’ evaluations. Second, the kindergarten-related variables jointly explained 11.2% of variation in the total score. The job position was a negative predictor of teachers’ evaluations, while school tenure and kindergarten ratings were positive. Finally, the urbanicity variable jointly explains 17.4% of the variation in the total score, which was the highest percentage in this study and was a positive predictor of teachers’ evaluations.

The hierarchical regression analysis revealed that urbanicity is a critical predictor of the teachers’ perceived quality: teachers in the countryside and villages have a relatively lower evaluation than those in counties and urban areas. In addition, job position, school tenure, and kindergarten rating significantly impacted teachers’ evaluations and account for 11.2% of the variation. A high level of teacher evaluation was associated with lower job positions, school tenure, and higher-rated kindergartens. Finally, while the demographic variables had the lowest interpretation rate for teacher evaluations, teachers’ ages and educational backgrounds still had negative effects on evaluations: low levels of teacher evaluation were associated with older ages and higher education levels (see Table 5).

## 5. Discussion

The main objective of this study was to understand how Chinese kindergarten teachers view the quality of Puhui EES. We created, validated, and applied the PEESQ scale to achieve this aim. This section discusses the major findings.

### 5.1. PEESQ Is Reliable and Valid

This study has validated the newly developed PEESQ, and its three dimensions have been confirmed: service adequacy at the input, service balance at the distribution, and public service welfare at the output. This is an advancement of the existing evaluation model in China, which focuses solely on the input and output, such as the outcome of children’s development [38]. PEESQ pays more attention to the overall process evaluation of Puhui quality and invites teachers to provide more specific suggestions for policy makers. The items of the PEESQ are related to the input, distribution, and output of the educational resources and services, all closely related to the policy goals and rooted problems inherent in the EES. While the PEESQ was developed to assess the quality of the Puhui EES, its use is not limited to Chinese contexts. The PEESQ can be used by researchers in other countries and regions to examine teachers’ perspectives of universal EES quality and to improve service effectiveness. The PEESQ could also be utilized to assess teachers’ perspectives of universal EES in other countries by selecting culturally appropriate dimensions in the scale.

### 5.2. Teachers Appreciated the Quality of Puhui EES

In this study, the mean score of the total quality evaluation was 4.41 (the full score was 5). Notably, “the evaluation” asserts that the social evaluation score of parents, teachers, and others should be higher than 4.25 in an EES evaluation survey [39]. This study exceeded the standard (*M* = 4.41) and indicates that the goals for developing the Puhui EES in China since 2010 have been achieved to an acceptable degree. Specifically, we found that the teachers rated the construct of public service welfare the highest, implying their satisfaction with the convenience and accessibility of Puhui EES. In contrast, they rated the construct of service balance the lowest, indicating some imbalance in the Puhui EES quality.

However, we found that teachers were most dissatisfied with “teacher salary” and “educational funding”. This finding is consistent with the existing study [40], which found that kindergarten teachers were disappointed with their salaries. This implies that more efforts should be made to enhance the compensation of kindergarten teachers, and more funding support should be provided to help enhance the quality of Puhui EES.

### 5.3. Factors Influencing Teachers’ Quality Evaluation

We identified urbanicity as the critical predictor of Chinese teachers’ quality evaluation. This finding indicates that kindergarten teachers in urban areas and counties have more positive evaluations than those in villages and rural areas. This urban–rural gap stems from the historical urban–rural differences in socioeconomic indicators and EES quality. To close the urban–rural gap in EES development and quality, governments have tried “all means” to implement universalized EES in rural China since 2010. However, rural areas still lag behind urban areas, as repeatedly reported in previous studies [15,17]. Furthermore, the quality of Puhui EES in rural areas was compromised because of the limited resources and unlimited teacher–child ratio. For instance, a rural teacher was responsible for a class of 70 children, whereas six urban teachers shared this workload [41]. These rural–urban differences have disappointed rural teachers with the EES system and, accordingly, downgraded their quality rating. To enhance the total quality of Puhui EES, the educational authorities must make more efforts to narrow the rural–urban gaps in resources and finance and thoroughly solve the inequalities between rural and urban teachers.

Second, we found that kindergarten ratings can also predict teachers’ quality evaluation. In particular, the teachers from third-class kindergartens scored significantly lower than those from other classes. This finding is consistent with the previous studies, which found that higher-rated kindergartens might receive more financial support and achieve higher quality; thus, these kindergarten teachers might have a higher positive evaluation of Puhui EES quality [14,40]. Moreover, compared with teachers from lower-rated kindergartens, teachers from higher-rated kindergartens might obtain higher salary guarantees and have a higher degree of social evaluation and status, which affects their evaluation of the Puhui EES quality. 

Third, we found that job positions and school tenure can predict teachers’ evaluations. In particular, the teachers with higher job positions tended to make negative evaluations. On the one hand, this could be because higher positions require more effort, workload, and labor; thus, the teachers tend to be more exhausted and disappointed. On the other hand, it could also be that the higher position made them knowledgeable of the quality problems. Therefore, future studies should be conducted to confirm the real causes. Nevertheless, the finding that the teachers with school tenure tended to make positive evaluations is consistent with existing studies [42,43], as they have more security and stability and tend to evaluate the quality of Puhui EES positively. Therefore, it is necessary to improve the construction of China’s job position and school tenure systems to promote the quality of kindergarten teachers and the Puhui EES.

## 6. Conclusions

This is the first nationwide survey of kindergarten teacher evaluations of Puhui EES quality. First, the results indicate that the PEESQ scale is reliable and valid, with three dimensions confirmed: service adequacy, service balance, and public service welfare. Second, Chinese teachers appreciated the quality of Puhui EES, with the construct of public service welfare ranked the highest, followed by the constructs of service adequacy and service balance. Third, urbanicity positively predicted teacher evaluations after controlling for demographic and kindergarten factors.

Still, this national study has several limitations. Firstly, the surveyed population only included kindergarten teachers and did not include other relevant stakeholders, such as educational administrators, parents, and children. Secondly, the children’s development outcome was not adopted to indicate Puhui EES quality. Thirdly, a multidimensional evaluation, instead of sole reliance upon questionnaire data, should be conducted to avoid response biases such as the social expectation effect from different populations. Fourthly, since there is no pre-existing, authentic, or reliable Puhui EES quality assessment system, we could only examine the scale’s content and construct validities, leaving the criterion-based validity unexplored. Finally, the present study indicated that several factors might influence teacher evaluation of the Puhui EES quality. Further studies are needed to verify and examine such findings.

Nevertheless, with a validated scale, this is the first national survey study to explore teachers’ perspectives of Puhui EES quality in China. Its findings have many implications for policy making in China and beyond. The most noteworthy implication is that this study extends the framework of research on the quality of EES and provides useful tools to evaluate universal service quality. Since the ultimate purpose of the scale’s development is to explore how to achieve available, affordable, quality EES, it is valuable to decision makers worldwide who are dedicated to promoting universal EES [3,15,44]. The second implication is that the study revealed that disadvantaged populations, such as kindergarten teachers without school tenure, are not financially stable. It is thus necessary to increase these teachers’ salaries to ensure the effective, balanced, sustainable, and high-quality development of the Puhui EES. Finally, there are significant differences in quality between rural and urban areas and between kindergartens of different ratings, which warrants more public attention, further studies, and policy changes.

## Figures and Tables

**Table 1:** Participants’ characteristics (*N* = 994).

Demographic Characteristics	*N*	%
Age		
<20	563	56.6%
20–29	291	29.3%
30–39	112	11.3%
≥40	28	2.8%
Teaching experience		
<2	448	12.8%
3–5	316	45.1%
6–8	102	31.8%
9–15	39	10.3%
≥16	89	9.0%
Educational background		
Junior high or below	42	4.2%
Senior high	381	38.3%
Junior college	422	42.5%
Undergraduate or above	149	15.0%
School tenure		
Yes	424	42.7%
No	570	57.3%
Job position		
Ordinary teacher	768	77.3%
Monitor	34	3.4%
Grade leader	58	5.8%
Director	49	4.9%
Principal	85	8.6%
Kindergarten rating		
Exemplary KG	688	69.2%
First-class KG	183	18.4%
Second-class KG	68	6.9%
Third-class KG	55	5.5%
Kindergarten type		
Public KG	795	80.0%
Puhui private KG	199	20.0%
Geographical area		
Urban city	234	23.6%
County	477	48.0%
Village	203	20.4%
Countryside	80	8.0%

Note: KG = kindergarten.

**Table 2:** Exploratory factor analysis results.

Item	Factor Loading			Commonality
	Service Adequacy	Service Balance	Public Service Welfare	
Q1	0.72			0.57
Q2	0.73			0.57
Q3	0.69			0.56
Q4	0.83			0.70
Q5	0.85			0.72
Q6	0.84			0.74
Q7	0.87			0.79
Q8		0.85		0.76
Q9		0.85		0.78
Q10		0.87		0.77
Q11		0.81		0.66
Q12		0.88		0.78
Q13		0.87		0.78
Q14		0.86		0.75
Q15		0.88		0.81
Q16		0.86		0.79
Q20		0.80		0.81
Q29		0.81		0.75
Q17		0.85		0.69
Q18			0.86	0.64
Q19			0.83	0.77
Q21			0.85	0.81
Q22			0.89	0.77
Q23			0.87	0.73
Q24			0.85	0.71
Q25			0.83	0.67
Q26			0.80	0.73
Q27			0.84	0.73
Q28			0.82	0.71
Q30			0.80	0.72
Q31			0.84	0.72
Q32			0.83	0.69
Total Explained Variance				72.39%

Note: The extraction method was principal axis factoring with a direct oblique rotation; rotation converged after 9 iterations.

**Table 3:** Item means, standard deviations, and reliability of PEESQ.

Construct	*M*	*SD*	Cronbach’s Alpha (α)	Split-Half Reliability
Service adequacy	4.46	0.64	0.91	0.82
Service balance	4.29	0.75	0.97	0.90
Public service welfare	4.50	0.61	0.97	0.80
Overall reliability	4.41	0.63	0.98	0.90

**Table 4:** Correlation matrix.

Variables	1	2	3	4	5	6	7
1. Total teacher evaluation score	——						
2. Age	−0.116 **	——					
3. Educational background	−0.009 **	−0.332 **	——				
4. Job position	−0.241 **	0.396 *	−0.108 **	——			
5. School tenure	0.132 **	−0.004 **	0.332 **	−0.145 **	——		
6. Kindergarten rating	0.230 **	0.060 **	0.124 **	0.119 **	0.104 **	——	
7. Geographical area	0.191 **	0.090 **	−0.215 **	0.103 **	0.207 **	0.255 **	——

Note: * *p* < 0.05, ** *p* < 0.01.

**Table 5:** Summary of hierarchical regression analysis predicting teacher evaluation.

Variable	β	*R* ^2^	Δ*R*^2^	F Value
Step 1		0.016	—	8.02 ***
Age	−0.133 ***			
Educational background	−0.053 ***			
Step 2		0.116	0.112	25.95 ***
Age	−0.039 ***			
Educational background	−0.030 ***			
Job position	−0.185 ***			
School tenure	0.119 ***			
Kindergarten rating	0.222 ***			
Step 3		0.18	0.174	30.98 ***
Age	−0.05 ***			
Educational background	−0.032 ***			
Job position	−0.162 ***			
School tenure	0.104 ***			
Kindergarten rating	0.148 ***			
Geographical area	0.215 ***			

Note: *** *p* < 0.001.

## Data Availability

The data presented in this study are available on request from the corresponding author. The data are not publicly available due to ethical requirements.

## Appendix A. Puhui Early Education Service Quality Scale

**Dimension**	**Definition**	**Indicators**
Service adequacy	Service adequacy refers to the resource investment adequacy of the Puhui EES.	Child–teacher ratio
		Percentage of teachers with ECE qualification certificate
		Percentage of degree holders
		Building area per child
		Outdoor activity area per child
		Picture books per child
		Toys and teaching aids per child
Service balance	Service balance refers to the resource distribution balance of the Puhui EES.	Financial investment for early childhood education
		Early childhood education funding per child
		Special funding for early childhood education
		Staff investment
		Salary guarantee for disadvantaged teachers
		Balanced development of urban and rural kindergartens
		Expand Puhui resources in rural areas
		Financial support for rural kindergartens
		Balanced development of different types of kindergartens
		Support for Puhui private kindergartens
		Support for disadvantaged kindergartens
		Governance of kindergartens in urban communities
Public service welfare	Public service welfare refers to the output benefits to children and society of the Puhui EES.	Scientific education and care activities
		Effective teacher–child interaction
		Provide family parenting
		Convenient transportation to kindergartens
		Equal admission of children with local household registration
		Affordable for most local families
		Suitable pick-up time in line with parents’ normal working hours
		Gross enrollment rate for children aged 3–6
		Percentage of children in public kindergartens
		Address the “3A” problems
		Coverage rate of Puhui kindergartens
		Construct public kindergartens in townships
		Support for children from low-income families
